# Assessing and Evaluating the Scope and Constraints of Idylla Molecular Assays by Using Different Source Materials in Routine Diagnostic Settings

**DOI:** 10.3390/ijms232012515

**Published:** 2022-10-19

**Authors:** Sanga Mitra Boppudi, Stefanie Scheil-Bertram, Elisabeth Faust, Anil Annamneedi, Annette Fisseler-Eckhoff

**Affiliations:** 1 Helios Dr. Horst Schmidt Kliniken Wiesbaden, Institute for Pathology and Cytology, 65199 Wiesbaden, Germany; 2 Gemeinschaftspraxis für Pathologie, 65199 Wiesbaden, Germany; 3 Team Biology of GPCR Signaling Systems (BIOS), CNRS, IFCE, INRAE, Université de Tours, Physiologie de la Reproduction et des Comportements (PRC), 37380 Nouzilly, France; 4 LE STUDIUM Loire Valley Institute for Advanced Studies, 45000 Orléans, France

**Keywords:** Idylla IVD assays, turnaround time, FFPE tissue sections, microsatellite instability, cytological fluids, isolated DNA, hematoxylin-eosin stained (HE) slides

## Abstract

For cancer treatment, diagnostics concerning tumor type and determination of molecular markers in short TAT is critical. The fully automated, real-time PCR-based molecular diagnostic Idylla assays are well established in many laboratories for qualitative detection, short TAT and routine screening of clinically relevant oncogenic mutations. According to the manufacturer, all IVD assays are recommended for use only with FFPE tissue samples of 5–10 µM dissections with at least 10% tumor content. In this study, we tested the performance and accuracy of the IVD assays along with the gene fusion assay (RUO) with different tissue/source materials like isolated DNA/RNA, cryomaterial, etc. The study also included testing archival FFPE tissue sections dating back from 20 years and a performance check for different pan-cancer samples individually. All the assays tested with FFPE sections and gDNA/RNA input showed above 96% accuracy and sensitivity, individually with 100% specificity. The Idylla assays also performed exceptionally well on the archival FFPE tissues, and the use of assays for other solid tumors was also remarkable. The performance test and accuracy of Idylla assays showed high efficiency with certain limitations. For the use of Idylla assays, both qualitative and quantitative applicability of different tumor source materials could produce efficient results in different diagnostic settings within a short TAT.

## 1. Introduction

Precision or personalized medicine (targeted therapy) is now gaining more importance in cancer treatment [1]. With the rapid development and increased use of technology, many new biomarkers have been identified until now, providing insights for targeted therapy of solid tumors. International guidelines now recommend performing next-generation sequencing (NGS) methods for detecting molecular aberrations, and also many laboratories have already incorporated NGS in their routine diagnostic settings. The detection of genomic alterations, which is still being done with respective gold standard techniques like FISH (fluorescence in situ hybridization), IHC (immunohistochemistry), RT-PCR (reverse transcription polymerase chain reaction), and pyro/Sanger sequencing, are being slowly replaced with NGS assays containing simultaneous variant detection in multiple genes and numerous samples, even though each technique has its pitfalls and advantages [2,3]. Therefore, simultaneous detection of either existing or novel genetic abnormalities in routine diagnostics now gained more importance as it can serve insights into differentiating histologically related tumors or presenting as important biomarkers for disease progression.

Moreover, the detection of many novel molecular alterations with NGS technologies also guides targeted therapy, providing a deeper understanding of the driver oncogenes and routing of molecular-based drugs [4]. Regardless of these advantages, NGS applications and analysis still remain a challenge. With the use of NGS in routine settings, several parameters come into question, like the cost per run/base, sample preparation time/cost, instrument run time/cost, personnel skills (technician/bioinformatician), percentage frequency of sequencing errors and overall efficiency with turnaround time.

However, for immediate patients’ treatment and diagnostics concerning tumor type, determining molecular markers or mutational status in a short turnaround time with high sensitivity and an economically acceptable method is very critical. For example, for patients with NSCLC to start immediately with the first-line treatment, EGFR tumor genotyping is essential, which also greatly impacts clinical management. If this data could be provided to the clinician within a day, the outcome of patient treatment would change drastically in regard to time. Such an alternative method is the fully automated, real-time PCR-based molecular diagnostic Idylla assays (Biocartis NV, Mechelen, Belgium). The system is a closed cartridge system containing all the necessary reagents for DNA isolation and PCR reaction with minimal hands-on time and automatic results generation. The Idylla molecular assays are well established in many laboratories now for qualitative detection, short turnaround time and routine screening of clinically relevant oncogenic mutations in EGFR, BRAF, KRAS, NRAS genes and MSI detection [5,6]. According to the manufacturer, all in vitro diagnostics (IVD) assays performed with the Idylla platform are recommended to be used only with formalin-fixed, paraffin-embedded (FFPE) tissue samples of 5–10 µM dissections with at least 10% tumor content and with considerable amount of minimum 50–600 mm² tissue area section to meet the Idylla tumor surface requirement.

In normal routine settings, the amount of sample material available is very much limited for performing different molecular tests covering entire diagnostic workflows. In such scenarios, the choice of molecular testing and the amount of material that should be used becomes the primary question. Sometimes different specimen types would be available from the patient, which cannot be used for routine diagnostic workflows or not intended to use by the manufacturer. There is little published data on the use of direct DNA input or stained cytological smears in the Idylla cartridges. These studies, however, were mostly related to the assessment of performance either in individual assays or more focusing on a specific tumor entity [6,7,8,9,10]. There are also a few studies where the use of Fine needle aspiration cytology (FNAC) was also performed with Idylla cartridges showing promising results with fast TAT but again limited to specific assay type or tumor type [11,12,13]. However, much uncertainty still exists about the relation between the use of other input materials and their performance in these Idylla assays.

This study attempts to address this issue, and we tested the performance and accuracy of Idylla assays with other different tissue/source materials, when available, like isolated DNA, cryomaterial, etc., along with the recommended FFPE material. Along with the IVD assays, the gene fusion (RUO version) cartridge has also been tested simultaneously with different materials. One purpose of this study was also to assess the extent to which these factors were performed in the assay cartridges when tested for different pan-tumor samples individually. This study also focused on the use of archival FFPE tissue sections in the cartridges dating back from 20 years. In this study, we assessed the sensitivity, specificity and limit of detection (LOD) of the Idylla molecular assays, and a combination of quantitative and qualitative approaches was used in the data analysis.

## 2. Results

Before actively starting the results and discussion part, some common terms and words should be defined for easy understanding of the outcome. From this point forward, the sample source type is defined as, A = FFPE-2 µM slide-scratched; B = HE slides-scratched; C = DNA input from FFPE scratched slide; D = DNA input from HE scratched slide; E = FFPE-5 µM slide-scratched; F = RNA input from FFPE scratched slide; G = RNA input from HE scratched slide.

Table 1 (CE-IVD assays) and Table 2 (GeneFusion Assay) provides an overview of the number of samples with their sample tumor type, tumor percentages, identified mutations and variant allele frequencies (VAF) from NGS or Pyromark methods and also stating the type of input material used for respective Idylla assays. The tables also provide data regarding the concentration and quality assessment of the isolated DNA (Table 1) and RNA (Table 2) either from FFPE or HE tissue slides, along with the amount of input material for each sample used in the cartridges. A total input of 5–25 µL of the isolated DNA/RNA was used for each molecular assay depending on the quality of the sample. Even though the quality of the samples was very poor, the amount of input material was not exceeded more than 25 µL.

### 2.1. Assessment of Sensitivity and Specificity of IVD-Labelled Assays

A total of 50 samples (including reference materials) were included in this study. For estimating the sensitivity and specificity of the IVD-Idylla molecular assays, a total of 25 samples were selected, comprising five samples per molecular assay and testing each sample for different source materials (A–E, where applicable) (Table 3 and Table 4). Table 3 provides the summary statistics for IVD-labelled assays (EGFR, KRAS, BRAF, and NRAS/BRAF) along with their performance in different source materials. The table also describes the concordance of the Idylla assays along with the target Cq obtained for the internal control of each cartridge. The results obtained from the analysis of MSI assays are summarized in Table 4, showing the target MSI score for each biomarker and what is striking about the figures in this table is the number of biomarkers amplified and the number of systems unstable for different source materials used.

**Figure 1:**
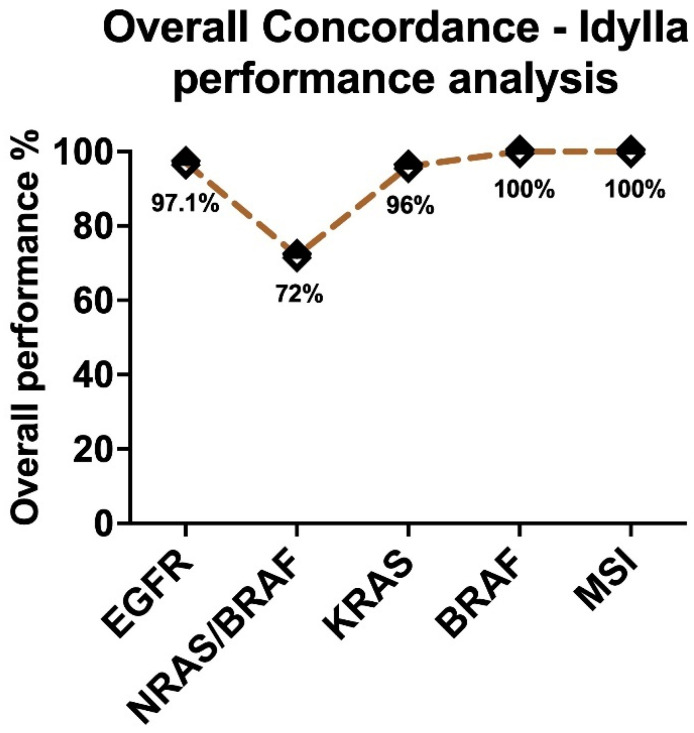
Graphical representation of the overall performance of the IVD-labelled Idylla molecular assays irrespective of the source material.

The single most striking observation to emerge from the data comparison was that there were no false positives reported in any assays, thereby showing a 100% specificity for every assay tested with respect to different materials. An overall assessment of the performance of the IVD-labeled molecular assays for all the source materials used in this study in concordance with other molecular testings was presented in Figure 1. The sensitivity of the assays, however, differed between assays, with a maximum of 100% for BRAF and MSI, and a higher sensitivity for EGFR (97.1%) and KRAS (96%). However, the sensitivity for NRAS/BRAF was only 72%, with the highest number of false negatives reported (13 out of 50 reported results). From the data (Table 3), we can see that only the NRAS/BRAF assay failed to replicate results in B (HE slides were scratched) and C (DNA from FFPE) sample types. Overall, these results indicate that the Idylla IVD assays showed a 93.1% sensitivity with 100% specificity, irrespective of the source materials.

### 2.2. Performance of Different Sample Types in the IVD-Assays

**Figure 2:**
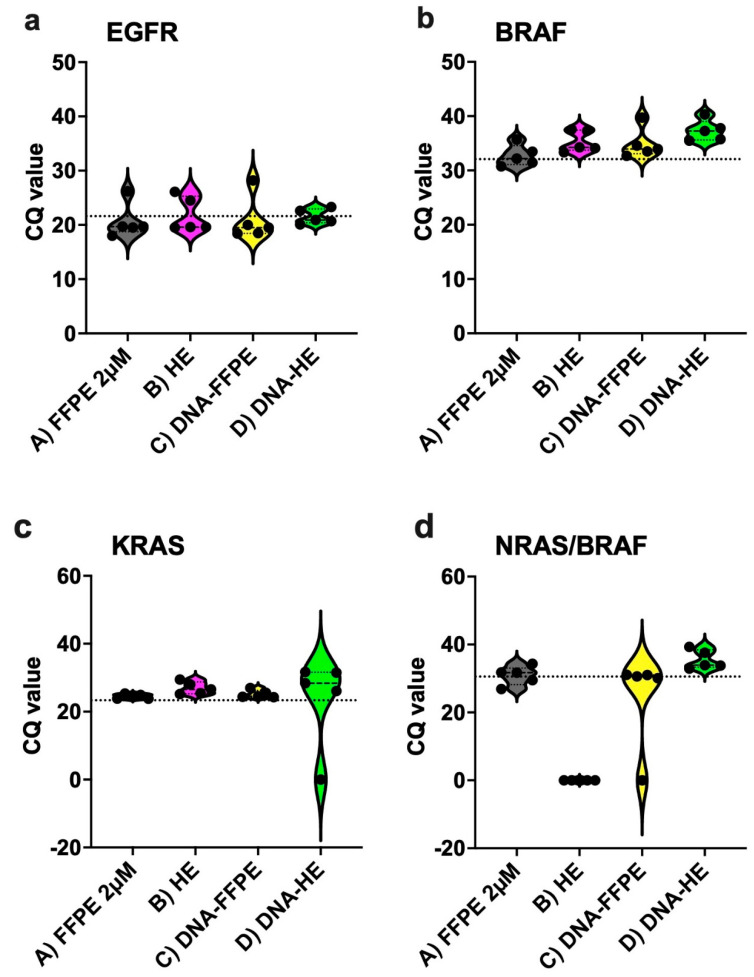
Summary graphs showing performance of the IVD−labelled assays and validation of different sample materials used ((**a**) EGFR, (**b**) BRAF, (**c**) KRAS, and (**d**) NRAS/BRAF). The dotted line in each figure panel indicates reference Cq values (FFPE 5 µM). EGFR = 21.60; BRAF = 32.39; KRAS = 23.70; NRAS/BRAF = 30.78.

**Figure 3:**
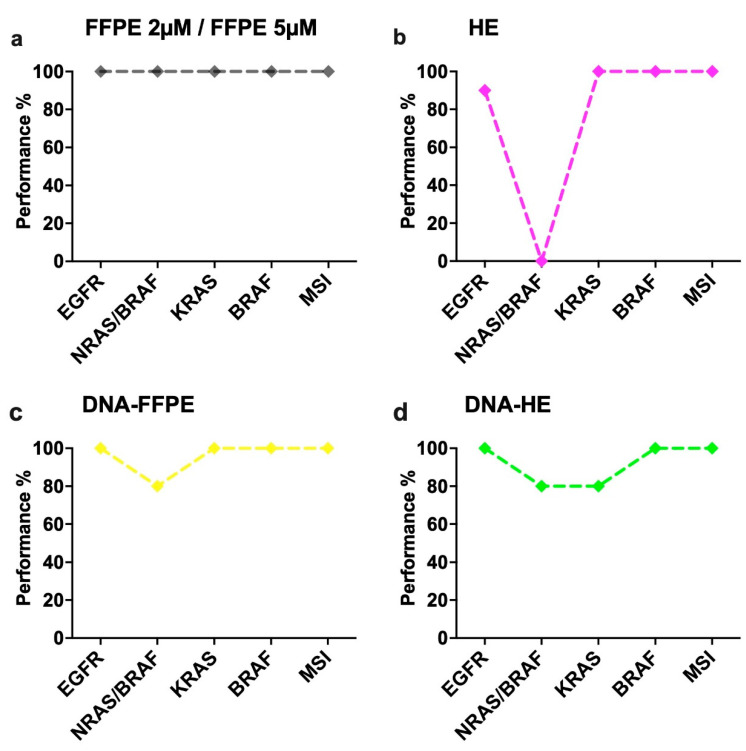
Evaluation of individual sample-type performance in different molecular assays. (**a**) Sample type A-FFPE 2 µM and E-FFPE 5 µM showed maximum performance (100%) between all the assays. (**b**) Sample type B-HE-tissue showed maximum performance in KRAS, MSI and BRAF (100%), slightly lower performance in EGFR (90%) and null performance in NRAS/BRAF assay (0%). (**c**) Sample type C-DNA-FFPE showed maximum performance among EGFR, MSI, KRAS and BRAF (100%), whereas lower performance in NRAS/BRAF (80%). (**d**) Sample type D-DNA-HE displayed maximum performance levels in EGFR, MSI and BRAF (100%) and lower performance levels among NRAS/BRAF and KRAS (80%).

Turning now to the experimental evidence, Figure 2 illustrates the summary statistics for each assay (EGFR; BRAF; KRAS; NRAS-BRAF) and its performance in each sample type in comparison to the Idylla recommend sample type E (5 µM FFPE section). The Cq values of sample type E were taken as a reference point for comparison. As shown in Figure 2, EGFR and BRAF performed exceptionally well in all sample types without much difference to the reference. While differential performance was seen in the KRAS assay with sample type D (DNA from HE) and in the NRAS/BRAF assay with sample type B (HE slides-scratched) and C (DNA from FFPE). From Table 4, the performance of the MSI assay can be compared with the reference material, also exhibiting a maximum performance rate. Figure 3 provides the breakdown of data with respect to different sample types in each assay. From the data in Figure 3, it is apparent that sample type A (2 µM FFPE section) and E had maximum performance in all of the IVD assays (100%), while sample type C and D had a slightly lower performance of 96% and 92%, respectively, in all of the IVD assays. The most striking result to emerge from the data is that sample type B showed the least efficiency, with only 78% overall.

### 2.3. Assessment of Sensitivity and Specificity of Gene Fusion Assay

Table 2 (gene fusion assay) provides an overview of the number of samples with their sample tumor type, tumor percentages, identified fusions with their fusion partners from NGS or FISH methods and also states the type of input material used for Idylla assay. For estimating the sensitivity and specificity of the GeneFusion Assay, a total of five samples were selected, and each sample was tested for different source materials (A, B, F, G, where applicable) (Table 5). Along with the five samples, a total of three predefined molecular reference standards (HD784, Seraseq FFPE v4 and CP-Positive Control) and two additional samples were used for monitoring the quality and performance. Table 5 provides the summary statistics for the GeneFusion Assay along with its performance in different source materials. The table also describes the concordance of the Idylla assays with alternative methods along with the values of target Cq of the specific fusion or ∆Cq Expression Imbalance-(3′–5′) or (3′-RNA controls) and also Cq of the RNA/DNA controls obtained for the internal control of each cartridge.

For the five samples tested, a total of four specific fusions and four expression imbalances should be detected by the Idylla assay. There were no false positives reported, thereby showing a 100% specificity for the assay tested with respect to different materials. The overall sensitivity of the assay with respect to different materials was only 87.5%. Of all the results, only one sample type (F -RNA from FFPE) failed to show the specific fusion, whereas two sample types F and G (RNA from HE), failed to show expression imbalance (Table 5), thus exhibiting 93.8% sensitivity for specific fusion detection and only 82% sensitivity performance for expression imbalance. For cases #41, #42 and #45 in sample type G, only the expression imbalances could not be detected because of the low quality of the RNA, which had DV 200 scores less than 50%, possibly explaining the cause of the failure and maybe with more input material in the cartridge, the expression imbalances could also be detected. For case #43, the sample type F (DV 200 score = 30%) failed completely, showing invalid results in which the DNA control has been properly amplified while the RNA controls have not, indicative of RNA degradation.

For the molecular reference standards, 100% sensitivity for specific fusion detection could be achieved, while only 67% sensitivity for expression imbalance.

- For HD784, all three specific fusions (ALK, ROS1, RET) were detected by specific PCR, and two-thirds expression imbalance (ALK, ROS1) could also be detected. The RET expression imbalance had a delta Cq (5′–3′) value of −0.58, which is on the borderline and the Cq of HKG is around 30 at the upper cutoff, meaning more input material is required for accurate analysis.
- For the CP-Positive control, ROS1 was detected both by specific detection and expression imbalance with a good input with HKG Cq at 28.6. In both of these controls, NTRK2/3 were invalid, and no borderline invalid curve was present, with a possible explanation that NGS controls mostly use artificial NTRK fragments, which cannot be detected by a full-length PCR design.
- For the Seraseq v4 material, all three specific fusions (ALK, ROS1, RET) along with MET Ex14 Skipping were detected by specific PCR, and the NTRK3 expression imbalance was also detected. The ALK expression imbalance could not be detected despite a high delta Cq (−5.29) value. The delta Cq cut-off is often dynamic and is increasing with high Cq HKG and is at a Cq 5′PCR of 38 at −5.3835. Thus, this sample tested represents a borderline negative case, and with the hypothesis of higher input, the assay would pick up the expression imbalance too. For Seraseq v4 material, the ROS1, RET and NTRK1 expression imbalance could not be detected, since, as mentioned in the instruction manual from Biocartis, the Seraseq version 4 reference material is an In-Vitro Transcript (IVT) RNAs with a sequence around the breakpoint of the fusion and doesn’t cover the 3′ regions, as a consequence, it is not suited for several of the expression imbalance detections by the GeneFusion Assay.

### 2.4. Determination of Repeatability of the Assays

Each sample type used in this study was tested for its performance by repeatability test (Supplementary Table S1). For each different assay, all sample types, one each (cases 2, 17, 12, 22 and 24 described in Table 1) were run three times under similar experimental conditions. For each sample, the coefficient of variation (CV%) was calculated using the Cq value of EGFR control/ NRAS Control/ KRAS target Cq value/ BRAF target Cq value in each cartridge. For MSI assays, the number of MSI biomarkers properly amplified was taken into account. For the GeneFusion Assays, case #41 (sample type A, F) and #42 (sample type B, G) was used, and the Cq value of the specific fusions was considered.

### 2.5. Performance of the Idylla Assays on Pan-Cancer Tissues

**Figure 4:**
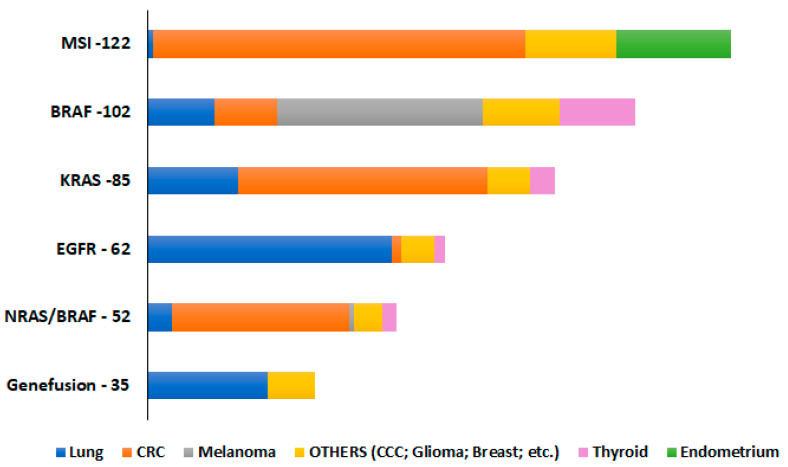
The overall number of pan-tumor samples, including other tumor entities (cholangiocellular carcinoma (CCC), prostate, gastric, small intestine, leiomyosarcoma, and pancreatic adenocarcinomas) tested for sample type A-FFPE 2 µM, to check their performance in different molecular assays.

The CE-IVD labels for Idylla molecular assay are assigned only to specific tumor types for each assay-NSCLC for EGFR; CRC for KRAS, NRAS-BRAF and MSI, and melanoma for BRAF. Even though the GeneFusion Assay is still a RUO version, the manufacturers state it has only been tested for NSCLC samples. To check the performance of these assays, different tumor entities were chosen and tested for each assay. For pan-cancer testing, only sample type A (FFPE-2 µM) was done due to the limitation of the costs of the assays. Other than the samples used in this study, all samples for which the routine diagnostics were performed in our lab from March 2021 to December 2021 were also considered (Figure 4), and Figure 4 shows the breakdown of the different pan-cancer samples tested for each assay. All the tumor entities evaluated showed 100% specificity for all the molecular assays tested with no false positives. The overall sensitivity of the assays for different tumor types was more than 95% for sample type A.

In this current study, different sample types (A–E) and along with different tumor entities could also be compared like for example, case #8 (thyroid), #10 (melanoma)- NRAS/BRAF assay; case #16, #18, #19 (thyroid)-BRAF assay; case # 9 (esophagus), #14 (lung), #12 (lung), #15 (pancreas)–KRAS assay and case #41 (oropharynx), #42 (thyroid)–GeneFusion Assay. An overall assessment of the performance of the pan-cancer samples for all the source materials tested in this study was a maximum of 100% for BRAF; 98% for KRAS, and 94% for the GeneFusion Assays. However, the sensitivity for NRAS/BRAF was only 82.5% since the highest number of false negatives were reported for sample type B. For the EGFR and MSI assays, all samples selected in this study belong to lung and CRC entities, respectively, so a comparison between sample types could not be achieved for them.

### 2.6. Performance of the Idylla Assays on Archived FFPE Tissues

For performing the analysis, always surgically resected tumors would not be available. Therefore, the availability of archival FFPE tissue material is one of the options for performing further analysis. The use of such tissues is absolutely formidable since, in most cases, the most tumor cells or high tumor content cells are present in the top four to five sections of the entire FFPE block, and, moreover, these blocks have been previously used for many routine diagnostic tests (so already cut for about three to four sections). Therefore, to check the performance of the IVD-labelled Idylla molecular assays on archival FFPE tissue material, tissue blocks that were approximately 20 years old (randomly from 2002 to 2022) were tested. For archival tissue testing, only sample type A (FFPE-2 µM) was done due to the limitation of the costs of the assays. From the samples listed in Table 1 and Table 2, the oldest tissue tested for each assay were EGFR-2012 (case #4), KRAS-2004 (case #11, Supplementary Figure S1), MSI-2010 (case #33), BRAF-2015 (case #35), NRAS-BRAF-2018 (case #37), gene fusion-2010 (case #50). Other timeline-prepared tissues, which are mentioned in Table 1 and Table 2, were also tested, and no significant correlation was found between the year of the fixed tissue and the outcome of the results. Other than the samples mentioned above, an additional fifteen archival samples were also tested for performance check (Supplementary Table S2). All the archival materials used in this study showed 100% specificity with no false positives. For sample type A, more than 94% of sensitivity was seen for the archival FFPE tissue, with very few samples showing invalid results in the first runs but when repeating the same samples with more amount of input of material revealed positive outcomes.

### 2.7. Performance of the Idylla Assays on Cytological Fluids

This study also set out with the aim of assessing the importance of the use of cytological fluids in EGFR (cases #32 and #33) and KRAS assays (cases #32 and #34). For cases, #32 and #33 (EGFR-WT), direct cytological fluid of 20 µL input was used in the cartridge, which resulted in EGFR positive results with Cq values of 15.9 and 26.7, respectively (Table 6).

For case #32 (KRAS-G12D with 3.1% VAF), direct cytological fluid of 20 µL input was used initially in the KRAS cartridge, which resulted in the wild-type. A second run was performed with the KRAS cartridge, now with 500 µL input of fluid which still emerged as a wild-type result. In both these instances for KRAS, when the raw data was observed a, very high Cq values were present for the G12D curves (Avg ~33.1), explaining the possibility of low LOD and low cell density in the sample. Furthermore, from this cytological fluid, DNA was extracted from directly using the fluid and from the paraffin-embedded cytology blocks in which the DNA concentration was too low in both samples when measured with Qubit DNA Broad range assay. The extracted DNA was then used in the KRAS cartridge with 40 µL input (direct) and 30 µL input (cytoblock) which resulted in a positive mutation detection with the KRAS total Cq values of 28.6 and 25.3, respectively. For case #34 (KRAS-G12D with 26.5% VAF), direct cytofluid pleural effusion (500 µL input) and DNA extracted from this effusion (40 µL input) were used to test the KRAS cartridge, and both resulted in KRAS positive mutation (G12D) with high Cq values of 28.9 and 30.8, respectively.

## 3. Discussion

This study set out with the aim of assessing the performance and accuracy of different Idylla assays with different sample materials other than the manufacturer’s recommended FFPE tissue samples of 5–10 µM. The sample materials used in this study include genomic DNA and RNA isolated from different source materials (FFPE tissues, cryomaterial/frozen tissue cuts and HE-stained slides), scratched tissue (non-deparaffinized/non-stained FFPE sections, HE slides), cryomaterial and tissue fluids and finally, 20-year-old archival FFPE tissue sections. All the source materials were collected from different tumor entities, including colorectal carcinoma (CRC), non-small cell lung cancer (NSCLC), papillary thyroid carcinoma (PTC), melanoma, oesophagus carcinoma, cholangiocellular carcinoma (CCC), pancreatic adenocarcinoma, etc. 

The availability of any kind of material from the tumor patient is very valuable since some of them cannot be reproduced or replaced. The cases selected for this study were only those availed with several materials, and due to this limitation, a total number of 50 cases were tested, which is not negligible. However, these investigations can be further supported with a large/greater number of cases by doing multicenter studies where large amounts of data would be available, and the limitations related to costs by an institution would also be shared equally. All the samples, including the references, were analyzed in the Idylla IVD molecular assays—EGFR, KRAS, BRAF, NRAS-BRAF and MSI. In principle, all the samples tested in these molecular assays showed similar concordance (>96% performance), except for NRAS-BRAF (72%) (Figure 1).

Among the different sample sources tested, the most obvious finding to emerge from the analysis is that FFPE (both 2 µM and 5 µM) material from various tumor entities displayed similar and high performance (100%) in all the Idylla IVD molecular assays (Figure 2 and Figure 3) and also for the GeneFusion Assays (Table 5). For each IVD molecular assay, the average Cq values from multiple case samples with FFPE material were akin to the reference Cq value (manufacturer’s recommendation). This is in line with previous studies showing the concordance and specificity of different assays included in our study [6,14,15,16]. Contrary to expectations, this study did not find a significant difference between the performance of the assays from sample types C and D (DNA isolated from FFPE tissue and HE-stained slides), showing an overall >91% sensitivity performance. This also accords with earlier observations, which showed that there is no significant difference in PCR amplification between DNA specimens from the unstained and HE-stained tissue [17,18]. They exhibited high performance in all the assays except for one sample each in the NRAS-BRAF cartridge and KRAS cartridge (Figure 3). This result may be explained by the fact that the two samples had poor-quality extracted DNA, resulting in high Cq values and invalid results. It is therefore important to bear in mind that results from such sample types should be interpreted with caution in the future in regard to sample quality and quantity. It is possible to hypothesize that these conditions are less likely to occur if more amount of input material (>25 µL DNA) is given for low-quality samples depending on the availability.

For the sample type B (HE-stained tissue material), high performance (100%) was displayed for the KRAS, BRAF and MSI assays, with comparatively limited performance in the EGFR assay (90%). One unanticipated finding was that there was a steep decline in performance, particularly in the NRAS-BRAF (0%) assays, showing invalid results for all the samples tested. Previous studies have shown a similar performance of HE material in Idylla EGFR assay [10]. The reason for this is not clear, but it may have something to do with the NRAS-BRAF assay itself being designed very stringent in which the quality of the sample is of utmost importance or increased DNA fragmentation in NRAS and BRAF loci [19]. In addition, this could be due to high background issues and heterogeneity, which are often associated with cytological specimens, including HE-stained tissue material [14,20]. However, when the raw curves were observed for case samples #6, #7, #8 and #10, the mutations to be identified were present with very high Cq values of more than 42. These results provide further support for the hypothesis that more amount of input material could resolve the issue. However, case #9 turned out to be a false negative result (wild-type) since it generally had very low-quality material as it failed in both extracted DNA and had very high Cq values for FFPE sample types.

On the question of HE-scraped tissue, this current study found that de-staining the specimen might help the outcome of the results in NRAS-BRAF assays. We have de-stained the HE slide of case #10 following the manufactures instructions (De-staining protocol available upon request from Biocartis). Upon de-staining, the Eosin stain was removed completely, still retaining the hematoxylin on the slide. This material was then scrapped and used directly in the NRAS-BRAF cartridge, which delivered a true positive result with 26.4 of the NRAS Cq value, which is in concordance with 2 µM FFPE sample. It is difficult to explain this result, but it might be related to the fact that the DNA integrity might be affected due to the staining procedure, and it is believed through previous observations that HE staining might also inhibit DNA amplification reactions [21]. However, the possible interference of tissue staining on DNA assays cannot be ruled out and, though this finding was unexpected, our study would suggest performing a de-staining protocol to tissue-stained slides/material for an outcome of better results when no other sample material is available. This argument can be further supported by the study conducted by Ercolani and colleagues [22], in which they showed that de-staining of the HE slides improved the mutation identification conditions for EGFR assays, thereby increasing the yield of amplified DNA or better fluorescence signals detection. 

However, in contrast to the direct use of an HE tissue sample or de-stained HE tissue, the DNA isolated from this tissue display a much better performance, comparatively, in all the assays (Figure 2 and Figure 3). Indeed, this is in line with previous systematic research showing high-quality performance using DNA isolated from archived HE-stained tissue and no interference of staining per se in a routine DNA diagnostic procedure [18]. Overall, this combination of findings provides some support for the conceptual premise that utilization of DNA isolated from HE-stained tissue is more appropriate instead of the direct de-stained tissue than the directly stained material.

For the MSI assays, contrary to expectations, this study did not find a significant difference between different materials tested and the outcome of the results, a 100% concordance has been observed. The only factor which varied between the materials tested, is the number of mutated/valid microsatellites detected. For example, in case #21, the Bethesda panel testing resulted in MSI high detection, with three out of ten markers being unstable.

In the IVD-Idylla MSI assay, all sample types resulted in MSI high detection with FFPE sample type (A and E) showing four of seven systems as unstable, whereas extracted DNA sample type (C and D), three of seven systems. For sample type B (HE-stained tissue material), only two out of the seven systems were detected as unstable. This study is in accordance with recent studies indicating that different source materials have been proven effective in successful findings of the Idylla MSI assays and also supporting the evidence of the use of the IVD MSI assays for different solid tumor types other than CRC [8,23,24,25]. One unanticipated finding was that for sample type B, the number of mutated/valid microsatellites detected and the number of biomarkers amplified was less than the other sample types, for example, cases #21 and #24. These results are consistent with the data obtained with other assays and the possible explanations or impacts of the use of direct HE material.

For the GeneFusion Assays, all the samples tested in this study had 100% specificity with an overall sensitivity of 96.3% for specific fusion detection and 80% for expression imbalance detection. For case #42, when 15 µL input was used for sample type G (RNA from HE scratched slide), the expression imbalance could not be detected, but when more amount of input material was used in the cartridge around 25 µL RNA, then the expression imbalance could also be detected. This here also supports the previous arguments that with low quality of the sample, more input material is recommended. For the GeneFusion Assays, through this study and during our normal routine diagnostics, a high number of invalid results happen to appear for NTRK1/2/3 expression imbalance detection. This result also corresponds to a recent study by [26] Chu et al., 2022, in which they show that NTRK1/2/3 rearrangements had the lowest sensitivity with only 81% (22/27). In the future, these results need to be interpreted with caution for the RUO version of the Gene fusion assay. Biocartis has announced that the CE-IVD version of the Idylla™ GeneFusion panel is planned for the end of 2022 with no NTRK1/2/3 rearrangements included in this version.

For checking the sensitivity and specificity performance of the use of direct DNA in all the IVD assays, case #40 (Reference control, CP-Positive control DNA) was tested, which had a DNA concentration of 6.25 ng/µL and 10 µL was used as input amount for each cartridge. For all the IVD assays tested, a 100% sensitivity and specificity were observed for this sample (Table 6). This study indicates that a minimum of 8 ng/µL gDNA concentration is required with at least 10 µL input (80 ng) to ensure the certainty of the results. It is also interesting to note that in all cytological samples of this study, the extracted DNA from such samples showed promising results. Hence, it could conceivably be hypothesized that with good quality DNA and with appropriate amount of input material, direct DNA can be used as alternative source material for all of the Idylla assays, which is in line with previous studies [8,27], but one should always remain with caution during data interpretation.

As mentioned earlier, the tumor cell percentage and the tumor cell content play a vital role in determining the results for the Idylla assays [7]. Another important finding from this study supporting this argument was with case #2, a NSCLC sample with only 30–40% tumor and very low cell quantity. The variant allele frequencies of EGFR mutations for this sample were determined by NGS method: Ex20:p.T790M (AF = 8.4%), Ex20:p.C797S (AF = 13.2%), Ex20:p.V802F (AF = 9.9%); and Ex21:p.L858R (AF = 29.7%). Only the two hotspot, therapy-relevant mutations could be detected and reported by Idylla EGFR assay in different sample types. When only one slide of unstained FFPE was used as input material, the T790M mutation was not reported by the system and also had a high Cq value of 25, which was significantly above the recommended threshold for EGFR. But when the test was repeated with three slides of FFPE material, both the mutations could be detected with a 19.7 Cq value. In the same case, when one slide of the stained HE slides was used as input material, surprisingly, the p.L858R mutation could not be detected even with a good Cq threshold of 19.5, for which the reason is not clear. This finding broadly supports the work of other studies in this area, linking the use of more input material for samples not meeting the defined criteria.

One other interesting finding in this study is case #11 with two positive KRAS mutations (p.G12D; p.G12V). The tissue used here was a multi-tissue block with eight different samples generated with two different mutations (four samples of p.G12D; three samples of p.G12V and one wild-type) (Supplementary Figure S1). In all of the sample types tested in this study, Idylla picked up G12D but not G12V (probably due to the variable VAF% of the mutations). The KRAS total Cq values (<22) indicate that the amount of amplifiable DNA in the cartridge was sufficient. The estimated AF of the G12V mutation in this run based on the raw data is most likely around 3% (or even lower), which is below the confirmed LOD of the test for G12V and the Cq values are very high (Average ~31.8), which causes a decrease of sensitivity. Similar to the above case, another sample in this study was case #30, with two KRAS mutations (p.G12D; p.G12C), and in the assay cartridge, both sample types A and B were used in combination in which only the G12D was picked up by the Idylla system due to its high VAF%. From the raw data, the G12C was present but with a high Cq value (Avg ~29.1) and lower LOD, resulting in this mutation not being reported. Here as well, by increasing the input, the G12V/C will most probably also be positive by the test. Moreover, the Idylla report only shows the most prevalent mutation in case there is no clinical relevance to report multiple mutations. Moreover, with this experiment, one can hypothesize that the combined use of multiple tissues or a combination of different sample types could result in promising outcome of results when the sample availability is scarce.

## 4. Materials and Methods

### 4.1. Sample Selection

A total of 50 samples (including reference materials) were included in this study. The study was conducted on a minimum of five samples for each molecular assay, which had previously confirmed SNVs/INDELS/Fusions with other orthogonal assays. The samples belong to different tumor entities like colorectal cancer (CRC), non-small cell lung cancer (NSCLC), melanoma and thyroid cancer etc. (Table 1). For each sample, the FFPE sections and hematoxylin-eosin stained (HE) slides were prepared using the standard procedures in everyday diagnostic pathology labs. The tumor cell content and the neoplastic cellularity estimation of each FFPE tissue was determined after careful examination by a molecular pathologist on the HE slides. The circled tumor area on the HE-slide was selected for the molecular analysis, where each tissue was macro-dissected. The tumor content in the current study samples had varying tumor percentages, from a minimum of 20% to a maximum of 95% (Table 1).

### 4.2. Sample Source Materials

In this current study, the different source materials tested and used as input material in the assay cartridges were: (1) genomic DNA (gDNA) and RNA isolated from FFPE tissues, cryomaterial, frozen tissue cuts, and HE-stained slides; (2) tissue scratched from non-deparaffinized and non-stained 5 µM and 2 µM FFPE sections; (3) tissue scratched from HE/PAS-stained slides; (4) direct input of the cryomaterial fluid or tissue section fluid; (5) archival FFPE tissue sections dating from 20 years back, and (6) to check the performance of the Idylla assays, all were also tested for different pan-tumor samples individually.

### 4.3. Molecular Reference Standards

In this study, for monitoring the quality and performance of the Biocartis Molecular assays, a total of three predefined molecular reference standards were purchased and used.

- Two targeted FFPE RNA fusion reference standards—HD784 (Horizon Discovery Inc., Cambridge, UK) and Seraseq FFPE tumor fusion RNA reference material v4 (SeraCare Life Sciences, Milford, MA, USA).
- One CP-positive control (PC; PC-RNA/PC-DNA), which is included in AmoyDx® HANDLE NGS Classic Panel (Amoy Diagnostics Co., Ltd., Xiamen, China).

The descriptions of each reference standard with their respective clinically relevant somatic variants (SNVs/INDELS/Fusions/CNV) are described in detail on the respective manufacturer’s website.

### 4.4. Nucleic Acid Isolations and Quantification

gDNA and RNA were extracted from the samples and reference standards (FFPE curl RNA- HD784; Seraseq v4) using the Maxwell^®^ FFPE Plus DNA Kit and Maxwell^®^ RSC RNA FFPE Kit, respectively (Promega, Madison, WI, USA). The DNA/RNA extraction was done as per the manufacturer’s instructions with an extra overnight incubation digestion step with proteinase K at 70 °C for DNA isolation. All extractions performed with the Promega kits were used on the semi-automated Maxwell RSC device (Promega, Madison, WI, USA) according to the manufacturer’s instructions. The DNA/RNA concentrations were determined using the Qubit^®^ dsDNA BR (Broad-Range; ThermoFisher Scientific, Waltham, MA, USA) Assay Kit and Qubit^®^ RNA BR Assay Kit, respectively, on the Qubit^®^ 4.0 Fluorometer (ThermoFisher Scientific, Waltham, MA, USA). All samples were stored in nuclease-free water or low TE at −80 °C for the prevention of the degradation of samples, especially RNA. The CP-Positive controls provided in the HANDLE NGS Classic Panel Kit had DNA concentration of 6.25 ng/µL and a RNA concentration of 4 ng/µL, according to the manufacturer.

The quality control of the isolated DNA and RNA was further checked using the 2100 Bioanalyzer system (Agilent, Santa Clara, CA, USA). The total RNA was quantified using the Agilent RNA 6000 Nano Kit, whereas the DNA was with the Agilent DNA 1000 Kit. The bioanalyzer assays and analyses were performed as instructed by the manufacturer. For the RNA quality check, the DV200 score was used to reliably classify the level of degradation of the sample.

### 4.5. Idylla Platform–Molecular Assays

Mutation detection for EGFR, KRAS, BRAF, NRAS-BRAF, MSI (CE-IVD versions) and gene fusion (RUO Version) was conducted on the automated Idylla platform with their respective cartridges, as instructed by the manufacturer for the FFPE sections and HE sections. The scraped tissue material was sandwiched between two wetted filter papers, which were placed directly in the corresponding cartridge. The cartridge was then loaded into the instrument for testing by fluorescent-based PCR amplification. The platform analyses and interprets the data generating a report on whether a mutation or group of mutations is identified or not. The Idylla samples were analyzed using the Idylla Explore analysis software (V.4.0). Depending on the concentration and quality of the isolated gDNA or RNA from FFPE or HE slides, a considerable amount of DNA/RNA is directly pipetted onto the filter paper and then placed in the cartridge. For the cryomaterial, the fluid is directly suspended in the cartridge.

The Idylla software gives a Cq (quantification cycle) value for every valid PCR curve. If the ΔCq value (difference between measured Cq values for mutant and wild-type PCR signals) is within the accepted range, the sample is termed as positive for the mutation, indicating the specific mutation/s identified. When the samples have valid wild-type Cq values, but the ΔCq value is not within the validated range, then it is termed as negative for the mutation (wild-type or No mutation detected). Sometimes, the results are interpreted as Invalid test cartridges, which might be due to various reasons like not enough tumor cell percentage (TC%) of a specimen, insufficient DNA input (tumor cell quantity), presence of inhibitors in the sample or cartridge malfunctioning.

### 4.6. Alternative Analytical Methods

In this current study, we validated the mutations with either of the four different commercially available NGS panels, AmpliSeq Focus panel or TruSight Tumour 15 panel (Illumina Inc., San Diego, CA, USA), Solid Tumor Plus Solution Kit (Sophia Genetics, Saint-Sulpice, Switzerland) and AmoyDx^®^ HANDLE NGS Classic Panel (Amoy Diagnostics Co., Ltd., Xiamen, China) which simultaneously could detect SNVs, INDELS, and gene fusions across pan-cancer solid tumors. The assays and analyses were performed as instructed by the manufacturer. Along with variant detections, the AmoyDx HANDLE NGS Classic Panel and Solid Tumor Plus Solution Kits also detect microsatellite instability (MSI). Paired normal tissue was not required as a control for analysis of MSI status on these NGS panels. The SNVs/INDELs were also confirmed by Pyrosequencing with Qiagen PyroMark Q24 assays (CE-IVD therascreen EGFR, KRAS, BRAF and RAS extension pyro kits) on the PyroMark Q24 Instrument (Qiagen, Hilden, Germany). The assays and analyses were performed as instructed by the manufacturer.

The RNA fusions were further confirmed with the traditional fluorescent in situ hybridization (FISH) method. For FISH analysis, FFPE slides of 5 µM thickness were used with an initial heat denaturation step (72 °C for 10 min) followed by manual processing using the Paraffin Pretreatment and Post-Hybridization Wash Buffer (Abbott Molecular, Abbott Park, IL, USA) according to manufacturer’s instruction. The probes used for analysis were ZytoLight SPEC Dual Color Probes (cMET, RET; NTRK1/2/3); ZytoLight SPEC ROS-1 Dual break apart probe, Zytolight SPEC ALK/EML4 Tricheck TM probe (Zytovision, Bremerhaven, Germany). A minimum of 100 cells were enumerated for each analysis.

For MSI detection, PCR-based fragment analysis was conducted. The fluorescent multiplex PCR analysis was done for the reference panel containing ten markers, namely, the Bethesda panel (Bat-25; Bat-26; D2S123; D5S346; D17S2509) and additional panel (Bat-40; D10S197; D13S153; D18S58, and MycI [28]) and the capillary electrophoreses were performed on the ABI 3130 or SeqStudio Genetic analyzer (Applied Biosystems, Foster City, CA, USA), and the analysis was completed using GeneMapper software (Version 6, Applied Biosystems, Foster City, CA, USA). For the MSI-PCR, DNA was extracted from paired tumors and normal tissues of the same patient. A peak shift of the markers is compared between the normal and tumor tissues categorizing the status as MSI-H (if more than two markers are unstable); MSI-L (if only 1–2 markers are unstable); MSS (all markers are stable with no shifts of peaks between paired tissues).

### 4.7. Data Analysis

Data were analyzed, and graphs were plotted using Microsoft Office Excel (version 16.54) and GraphPad Prism (version 9). Overall performance for each molecular assay was calculated based on the presence or absence of mutation detected by the Idylla test in individual samples among different sample types, and the average score was assessed. The comparative performance of each sample type between the molecular assays was calculated as described above.

## 5. Conclusions

The purpose of the current study was to determine the sensitivity and specificity performance of the Idylla assays when different source materials were used. This appears to be the first study to compare the experiences of the use of different materials in all available Idylla assays (until now). This study is also the only empirical investigation into the impact of the GeneFusion Assays with regard to the use of HE-stained slides or the use of direct RNA from such materials. Our study has shown that 2 µM FFPE sections also provide the highest quality results in comparison to the recommended sample requirements. The findings of this investigation complement those of earlier studies in regard to the use of direct DNA input in the cartridges either from FFPE sections or HE-stained slides or cytological fluids. Notwithstanding the relatively limited sample, this work offers valuable insights into following the de-staining procedure of the HE slides prior to use and also suggests that the utilization of DNA isolated from HE-stained tissue is more appropriate instead of the direct de-stained tissue than the direct stained material. The study has also shown that pan-cancer samples or archival FFPE samples can be tested with all the assays with very assuring results. 

To conclude, the results of this investigation, although preliminary, show that in principle promising results could be achieved with different materials when used with good quality and with appropriate amount of input material for Idylla assays. Thus, confirming the robustness of the Idylla system providing critical clinical implications in short time. This research has thrown up many questions in need of further investigation of possible performance interferences for stained sample materials. Further research could also be conducted to determine the effectiveness of LOD when using such alternative materials.

## Figures and Tables

**Table 1:** Characteristics of selected samples with mutation status detected by alternative analyses for Idylla IVD assays.

Case No. #	Year of Tissue Prepared	Tumor Cells (%)	Cancer Type	Previous Result	VAF %	Alternate Method (s)	Idylla Cartridge Used	Input Material (s) in Cartridge	gDNA Input-FFPE Scratched Slide			gDNA Input-HE Scratched Slide		
									DNA Conc. (ng/μL)	DNA Quality Score *	DNA Input in the Cartridge-µL	DNA Conc. (ng/μL)	DNA Quality Score *	DNA Input in the Cartridge-µL
1	2019	90%	NSCLC	EGFR-Ex19:p.L747_S7522del	38.9%	NGS; Pyro	EGFR	FFPE; gDNA	26.4	1	10	34.7	2	15
				EGFR-Ex19:p.A755G	39.4%									
2	2020	30–40%	NSCLC	EGFR-Ex20:p.T790M	8.4%	NGS; Pyro	EGFR	FFPE; gDNA	24.1	1	10	23.2	3	25
				EGFR-Ex21:p.L858R	29.7%									
3	2020	70%	NSCLC	EGFR-Ex 20:p.S768I	31.6%	NGS; Pyro	EGFR	FFPE; gDNA	54.2	1	15	70.5	3	20
				EGFR-Ex21:p.L858R	14.8%									
4	2012	50%	NSCLC	EGFR-Ex 19: p.E746_T753>V	51.5%	NGS; Pyro	EGFR	FFPE; gDNA	31.6	2	20	29.1	3	20
5	2021	75%	NSCLC	EGFR-Ex19: p.L747_T751del	34.4%	NGS	EGFR	FFPE; gDNA	143.5	1	10	52	2	15
6	2020	80%	CRC	NRAS-Ex2-c:49delA	28.40%	NGS	NRAS/BRAF	FFPE; gDNA	91.3	1	10	38.2	2	20
				BRAF-Ex15:p.V600E	41.70%	NGS	NRAS/BRAF	FFPE; gDNA			10			20
				MSI High (5/10 System Unstable)		NGS; FA	MSI	FFPE; gDNA			10			20
7	2019	80%	CRC	NRAS-Ex 3:p.Q61L BRAF-WT	22.10%	NGS; Pyro	NRAS/BRAF	FFPE; gDNA	139	1	12	21.3	3	20
8	2019	100%	PTC	NRAS-Ex 3:p.Q61R BRAF-WT	34.20%	NGS; Pyro	NRAS/BRAF	FFPE; gDNA	36	2	20	88.8	2	15
9	2019	80%	CRC	NRAS-Ex 3:p.Q61R BRAF-WT	20.20%	NGS; Pyro	NRAS/BRAF	FFPE; gDNA	12.4	4	25	24.5	4	25
10	2020	95%	Melanoma	BRAF-Ex15:p.V600E, NRAS-WT	14.40%	Pyro	NRAS/BRAF	FFPE; gDNA	275	1	5	35.3	4	25
11	2004	70%	CRC	KRAS-Ex2:p.G12D	14.1%	Pyro	KRAS	FFPE; gDNA	196.1	1	12	50.9	1	15
				KRAS-Ex2:p.G12V	2.1%									
12	2019	30–40%	NSCLC	KRAS-Ex 2:p.G12A	26.1%	NGS; Pyro	KRAS	FFPE; gDNA	101.1	1	10	33.5	1	15
13	2019	80%	Oesophageal Carcinoma	KRAS-Ex 2:p.G13D	55.94%	NGS; Pyro	KRAS	FFPE; gDNA	86.5	1	10	64.7	3	25
14	2018	65%	NSCLC	KRAS-Ex4:p.Ala146Thr	63.86%	NGS; Pyro	KRAS	FFPE; gDNA	295.1	1	5	9.7	2	25
15	2022	60%	Pancreatic cancer	KRAS- Ex 2:p.G12R	9.50%	Pyro	KRAS	FFPE; gDNA	59.6	1	12	167.2	1	10
16	2021	90%	Thyroid cancer	BRAF-Ex15:p.V600E	19.70%	Pyro	BRAF	FFPE; gDNA	10	1	15	13.2	1	10
17	2018	75%	Melanoma	BRAF-Ex15:p.V600K	32.90%	NGS; Pyro	BRAF	FFPE; gDNA	259	1	8	96.2	1	10
18	2021	50%	Thyroid cancer	BRAF-Ex15:p.V600E	36.70%	Pyro	BRAF	FFPE; gDNA	113.9	1	10	38.5	3	25
19	2021	90%	Thyroid cancer	BRAF-Ex15:p.V600E	24.10%	Pyro	BRAF	FFPE; gDNA	95	1	10	118.1	2	15
20	2022	50%	Melanoma	BRAF-Ex15:p.V600E	44.70%	Pyro	BRAF	FFPE; gDNA	45.3	2	15	43.8	2	20
21	2020	80%	CRC	MSI High (3/10 System unstable)		FA	MSI	FFPE; gDNA	190	1	8	8.9	1	15
22	2010	80%	CRC	MSS		FA	MSI	FFPE; gDNA	141	1	10	5.35	1	15
23	2021	70%	CRC	MSI High (7/10 System unstable)		FA	MSI	FFPE; gDNA	132	1	10	125.3	2	10
24	2020	90%	CRC	MSI High (8/10 System unstable)		NGS; FA	MSI	FFPE; gDNA	322	1	8	140.5	1	10
25	2017	30%	Melanoma	BRAF-WT		Pyro	BRAF	FFPE-HE	N/A	N/A	N/A	N/A	N/A	N/A
26	2021	60%	CRC	NRAS-WT; BRAF-WT		Pyro	NRAS-BRAF	FFPE; gDNA-HE	N/A	N/A	N/A	11.5	1	10
27	2021	70%	NSCLC	EGFR-Ex18: p.G721S	5.80%	NGS	EGFR	FFPE-HE	N/A	N/A	N/A	N/A	N/A	N/A
28	2020	90%	NSCLC	EGFR-Ex19: p.E746_S752delins	57.20%	NGS	EGFR	FFPE-HE	N/A	N/A	N/A	N/A	N/A	N/A
29	2021	80%	NSCLC	EGFR-Ex21:p.L858R	14.80%	NGS	EGFR	gDNA-FFPE	5.5	1	15	N/A	N/A	N/A
30	2022	80%	CRC	KRAS-Ex2:p.G12D KRAS-Ex2:p.G12C	25.8% 1.43%	NGS	KRAS	FFPE-HE + FFPE	N/A	N/A	N/A	N/A	N/A	N/A
31	2021	20%	Thyroid cancer	BRAF-Ex15:p.V600E	6.90%	Pyro	BRAF	gDNA-HE	N/A	N/A	N/A	6.7	4	10–25 µL
32	2021	60%	cholangiocellular carcinoma	EGFR-Ex19:PV742I	46.70%	NGS	EGFR	Cytological fluid; gDNA	20 µL Cytofluid material input in the cartridge					
							KRAS		20–500 µL Cytofluid material input in the cartridge and gDNA prepared from FFPE-cytoblock with 30 µL input and gDNA prepared from Cytofluid material with 40 µL input					
				KRAS:Ex2:G12D	3.10%									
33	2021	30%	NSCLC	EGFR-WT		NGS	EGFR	Cytofluid pleural effusion	20 µL Cytofluid material input in the cartridge					
34	2022	10%	IPMN	KRAS-Ex2: G12D	26.50%	Pyro	KRAS	pleural effusion fluid; gDNA	gDNA prepared from Cytopleural effusion-with 40 µL input					
35	2015	70%	Melanoma	BRAF-WT		Pyro	BRAF	FFPE	N/A					
36	2011	20%	CRC	MSS		FA	MSI	FFPE	N/A					
37	2018	50%	CRC	NRAS-WT; BRAF-WT		Pyro	NRAS-BRAF	FFPE	N/A					
38	2022		Oesophageal Carcinoma	MSS		FA	MSI	FFPE-HE + PAS	N/A					
39	2021	90%	NSCLC	KRAS-WT		NGS	KRAS	FFPE	N/A					
				EGFR-WT			EGFR	FFPE	N/A					
				NRAS-WT; BRAF-WT			NRAS-BRAF	FFPE	N/A					
40	N/A		CP-Positive Control (Reference Standard)	EGFR-Ex20: p.T790M EGFR-Ex19:p.E746_A750del EGFR-Ex21:p.L858R	7.46% 7.12% 8.42%	NGS	EGFR; BRAF; KRAS; MSI; NRAS-BRAF	gDNA	6.25 ng/µL–10 µL direct input in each cartridge					
				KRAS-Ex 2:p.G13D	20.89%									
				NRAS-WT BRAF-WT MSI-High										

CRC—colorectal carcinoma; PTC—Papillary thyroid carcinoma; NSCLC—non-small cell lung cancer; IPMN—Intraductal Papillary Mucinous Neoplasm; FFPE—formalin-fixed paraffin-embedded; Gdna—Genomic DNA; N/A—not applicable; VAF—Variant allele frequency; NGS—next-generation sequencing; Pyro—pyrosequencing; NE—not evaluated; Ex—Exon; WT—wild-type; conc—concentration; MSI—microsatellite instability; MSS—microsatellite stable; HE—hematoxylin-eosin-stained slides; FA—ABI Fragment analysis. * DNA quality score- Quality graded from 1 to 4, with 1 being the best.

**Table 2:** Characteristics of selected samples with mutation status detected by alternative analyses for Idylla GeneFusion Assays–use of GeneFusion-RUO cartridges.

Case No. #	Year of Tissue Prepared	Tumor Cells (%)	Cancer Type	Previous Result	Alternate Method (s)	Input Material (s)	RNA Input-FFPE Scratched Slide			RNA Input-HE Scratched Slide			RNA Input-Other Materials		
							RNA Conc. (ng/μL)	RNA Quality Score-DV 200	RNA Input in the Cartridge-µL	RNA Conc. (ng/μL)	RNA Quality Score-DV 200	RNA Input in the Cartridge-µL	RNA Conc. (ng/μL)	RNA Quality Score-DV 200	RNA Input in the Cartridge-µL
41	2020	30%	Neck level I-V -Oropharynx Carcinoma	ALK fusion ALK::EML4	NGS; FISH	FFPE; RNA	48.8	50–70%	12	14.7	30–50%	20	N/A	N/A	N/A
42	2020	70%	PTC	RET fusion RET::NCOA4	NGS; FISH	FFPE; RNA	350	>70%	6	46.4	50–70%	25	N/A	N/A	N/A
43	2020	60%	NSCLC	ALK fusion ALK::EML4	NGS; FISH	FFPE; RNA	20.4	30–50%	15	5.63	30–50%	25	N/A	N/A	N/A
44	2019	90%	NSCLC	MET Ex14 Skipping	NGS; FISH	FFPE; RNA	91.1	50–70%	12	48.5	30–50%	20	N/A	N/A	N/A
45	2019	80%	NSCLC	ROS1 fusion ROS1::SDC4	NGS; FISH	FFPE; RNA	12.5	30–50%	25	10	<30%	25	N/A	N/A	N/A
46	N/A	Horizon HD 784		ALK, RET, ROS1 fusions EML4::ALK, CCDC6::RET and SLC34A2::ROS1	NGS	RNA	N/A	N/A	N/A	N/A	N/A	N/A	18.2	>70%	5.8
47	N/A	SeraSeq V4 RNA reference material		ALK, RET, ROS1, NTRK3, NTRK1 fusions; MET Ex14 Skipping	NGS	RNA	N/A	N/A	N/A	N/A	N/A	N/A	10.2	>70%	9
48	N/A	CP-Positive Control RNA		ROS1 fusion SLC34A2::ROS1	NGS	RNA	N/A	N/A	N/A	N/A	N/A	N/A	4	>70%	13
49	2014	90%	NSCLC	ALK fusion ALK::EML4	NGS; FISH	FFPE	N/A	N/A	N/A	N/A	N/A	N/A	N/A	N/A	N/A
50	2010	50%	Leiomyosarkom	Wild-type	NGS; FISH	FFPE	N/A	N/A	N/A	N/A	N/A	N/A	N/A	N/A	N/A

FFPE—formalin-fixed paraffin-embedded; N/A—not applicable; NSCLC—non-small cell lung cancer; VAF—Variant allele frequency; NGS—next-generation sequencing; Pyro—pyrosequencing; WT, wild type; NE—not evaluated; RUO—research use only; FISH—Fluorescence in situ hybridization; Ex—Exon.

**Table 3:** Comparative analysis of individual sample-type performance in different IVD-molecular assays.

Case No. #	Alternative Method Result	Conclusion of Idylla Test-Mutation Detected YES/NO					Conclusion of Idylla Test-Cq Value−EGFR Control/NRAS Control/KRAS Target Cq Value/BRAF Target Cq Value					Concordance	Idylla Performance Analysis
		A	B	C	D	E	A	B	C	D	E		
EGFR													
1	EGFR-Ex 19: p.L747_S7522del	YES	YES	YES	YES	YES	18	19.6	19.5	20.7	18.5	YES	100%
	EGFR-Ex 19:p.A755G	N/A	N/A	N/A	N/A	N/A						N/A	
2	EGFR-Ex 20: p.T790M	YES	YES	YES	YES	YES	19.7	19.5	20	22.6	21.9	YES	90%
	EGFR-Ex 21:p.L858R	YES	NO	YES	YES	YES						Partial	
3	EGFR-Ex 20:p.S768I	YES	YES	YES	YES	YES	19.7	19.6	18.5	20.1	19.1	YES	100%
	EGFR-Ex 21:p.L858R	YES	YES	YES	YES	YES						YES	
4	EGFR-Ex 19: p.E746_T753>V	YES	YES	YES	YES	YES	26.2	26.1	28.2	23.3	29.8	YES	100%
5	EGFR-Ex 19: p.L747_T751del	YES	YES	YES	YES	YES	19.5	24.5	18.4	20.9	18.7	YES	100%
NRAS/BRAF													
6	BRAF-Ex 15:p.V600E	YES	NO	YES	YES	YES	29.5	0	31	32.9	28.4	Partial	90%
	NRAS-Ex 2-c:49delA	N/A	N/A	N/A	N/A	N/A						N/A	
7	NRAS-Ex 3-p.Q61L	YES	NO	YES	YES	YES	31.7	0	30.1	33.9	29.3	Partial	80%
	BRAF-WT	YES	NO	YES	YES	YES						Partial	
8	NRAS-Ex 3-p.Q61R	YES	NO	YES	YES	YES	31.8	0	31.1	33.8	30.5	Partial	80%
	BRAF-WT	YES	NO	YES	YES	YES						Partial	
9	NRAS-Ex 3-p.Q61R	YES	NO	NO	NO	YES	34.3	0	0	39.3	37.3	Partial	40%
	BRAF-WT	YES	NO	NO	NO	YES						Partial	
10	BRAF-Ex 15:p.V600E	YES	NO	YES	YES	YES	26.9	0	30.6	37.6	28.4	Partial	80%
	NRAS-WT	YES	NO	YES	YES	YES						Partial	
KRAS													
11	KRAS-Ex 2:p.G12D/V	YES	YES	YES	YES	YES	23.84	25.19	24.27	28.39	23	YES	100%
12	KRAS- Ex 2:p.G12A	YES	YES	YES	YES	YES	25.33	29.45	25.57	31.68	23.98	YES	100%
13	KRAS- Ex 2:p.G13D	YES	YES	YES	YES	YES	23.79	25.43	24.54	26.06	23.32	YES	100%
14	KRAS -Ex 4:p.A146T	YES	YES	YES	NO	YES	24.8	26.54	24.38	0	23.95	Partial	90%
15	KRAS- Ex 2:p.G12R	YES	YES	YES	YES	YES	24.93	28.01	26.98	31.5	24.28	YES	100%
BRAF													
16	BRAF-Ex 15:p.V600E	YES	YES	YES	YES	YES	35.69	37.30	39.72	40.31	32.5	YES	100%
17	BRAF-Ex 15:p.V600K	YES	YES	YES	YES	YES	33.47	37.57	34.57	37.77	33.56	YES	100%
18	BRAF-Ex 15:p.V600E	YES	YES	YES	YES	YES	31.43	34.25	32.7	37.26	32.4	YES	100%
19	BRAF-Ex 15:p.V600E	YES	YES	YES	YES	YES	30.75	34.12	33.5	35.47	32.3	YES	100%
20	BRAF-Ex 15:p.V600E	YES	YES	YES	YES	YES	32.19	33.35	33.96	35.76	31.2	YES	100%

A—FFPE-2 µM slide- scratched; B—HE slide- scratched; C—DNA Input from FFPE scratched slide; D—DNA Input from HE scratched slide; E—FFPE-5 µM slide-scratched; N/A—not applicable; Cq value—quantification cycle; Ex—Exon; WT—wild-type.

**Table 4:** Comparative analysis of individual sample-type performance in Idylla MSI assay.

Case No. #	Alternative Method Result	Input Material (s) in Idylla Cartridge	Idylla-Sample MSI Status	Idylla Quality Status	ACVR2A	BTBD7	DIDO1	MRE11	RYR3	SEC31A	SULF2	Concordance	Idylla Performance Analysis
6	MSI High (5/10 System unstable)	A	MSI High (6/7 System unstable)	7/7 biomarkers amplified	1	1	0.7	0.38	0.98	1	0.98	YES	100%
		B	MSI High (7/7 System unstable)	7/7 biomarkers amplified	1	1	0.64	0.52	0.96	1	0.91	YES	100%
		C	MSI High (7/7 System unstable)	7/7 biomarkers amplified	1	1	0.88	0.52	1	1	1	YES	100%
		D	MSI High (7/7 System unstable)	7/7 biomarkers amplified	1	1	0.73	0.75	0.99	1	0.98	YES	100%
		E	MSI High (7/7 System unstable)	7/7 biomarkers amplified	1	1	0.8	0.62	1	1	1	YES	100%
21	MSI High (3/10 System unstable)	A	MSI High (4/7 System unstable)	7/7 biomarkers amplified	1	0.92	0.79	0.4	0.15	0.15	0.62	YES	100%
		B	MSI High (2/7 System unstable)	6/7 biomarkers amplified	1	-	0.8	0.25	0.03	0.19	0.5	YES	100%
		C	MSI High (3/7 System unstable)	7/7 biomarkers amplified	1	0.89	0.74	0.32	0.12	0.13	0.33	YES	100%
		D	MSI High (3/7 System unstable)	7/7 biomarkers amplified	0.98	0.79	0.75	0.32	0.09	0.1	0.22	YES	100%
		E	MSI High (4/7 System unstable)	7/7 biomarkers amplified	1	0.92	0.79	0.4	0.15	0.15	0.62	YES	100%
22	MSS	A	MSS-No mutation detected (7/7 stable)	7/7 biomarkers amplified	0	0	0	0	0	0	0	YES	100%
		B	MSS-No mutation detected (7/7 stable)	7/7 biomarkers amplified	0	0	0	0	0	0	0	YES	100%
		C	MSS-No mutation detected (7/7 stable)	7/7 biomarkers amplified	0	0	0	0	0	0	0	YES	100%
		D	MSS-No mutation detected (7/7 stable)	7/7 biomarkers amplified	0	0	0	0.01	0	0	0	YES	100%
		E	MSS-No mutation detected (7/7 stable)	7/7 biomarkers amplified	0	0	0	0	0	0	0	YES	100%
23	MSI High (7/10 System unstable)	A	MSI High (7/7 System unstable)	7/7 biomarkers amplified	1	1	1	1	1	1	1	YES	100%
		B	MSI High (7/7 System unstable)	7/7 biomarkers amplified	1	1	0.99	1	1	1	1	YES	100%
		C	MSI High (7/7 System unstable)	7/7 biomarkers amplified	1	1	1	1	1	1	1	YES	100%
		D	MSI High (7/7 System unstable)	7/7 biomarkers amplified	1	1	1	1	1	1	1	YES	100%
		E	MSI High (7/7 System unstable)	7/7 biomarkers amplified	1	1	1	1	1	1	1	YES	100%
24	MSI High (8/10 System unstable)	A	MSI High (5/7 System unstable)	7/7 biomarkers amplified	0.98	0.37	0.02	0.95	0.98	1	0.99	YES	100%
		B	MSI High (5/7 System unstable)	6/7 biomarkers amplified	1	-	0	0.61	0.81	1	1	YES	100%
		C	MSI High (4/7 System unstable)	7/7 biomarkers amplified	1	0.26	0	1	0.21	1	1	YES	100%
		D	MSI High (4/7 System unstable)	7/7 biomarkers amplified	1	0.24	0.02	0.99	0.15	1	0.99	YES	100%
		E	MSI High (5/7 System unstable)	7/7 biomarkers amplified	0.99	0.22	0.01	0.98	0.67	1	1	YES	100%

A—FFPE-2 µM slide-scratched; B—HE slides-scratched; C—DNA Input from FFPE scratched slide; D—DNA Input from HE scratched slide; E—FFPE-5 µM slide-scratched; N/A—not applicable; MSI—microsatellite instability; MSS—microsatellite stable.

**Table 5:** Comparative analysis of individual sample-type performance in GeneFusion Assay.

			Conclusion of Idylla Test								
Case No. #	Alternative Method Result	Input Material (s) in Idylla Cartridge	Specific Fusion Detected YES/NO	Expression Imbalance Detected YES/NO	Cq Specific Fusion	∆Cq Expression Imbalance-(3′–5′) or (3′-RNA Controls)	Cq of the RNA Controls	Cq of the DNA Controls	Invalid Result /Comments	Overall Concordance	Idylla Performance Analysis
41	ALK fusion ALK::EML4	A	YES	YES	27.7	−6.4	26.5	27.9		YES	100%
		B	YES	YES	32.4	1.7	32.5	30.4		YES	100%
		F	YES	YES	29.4	−6.4	29.5	31.9		YES	100%
		G	YES	NO	32.7	−5.9	32.6	33.3	NTRK2-Invalid	Partial	50%
42	RET fusion RET::NCOA4	A	N/A	YES	N/A	−0.9	24.7	25.1		YES	100%
		B	N/A	YES	N/A	−1.2	26.8	27.1		YES	100%
		F	N/A	YES	N/A	−1.1	25.9	27.8		YES	100%
		G	N/A	YES	N/A	−1.1	29.3	30.5	NTRK1-Invalid	YES	100%
43	ALK fusion ALK::EML4	A	YES	YES	31.1	1.9	30.7	29.7		YES	100%
		B	YES	YES	33.6	2	33	31.4		YES	100%
		F	NO	NO	NO	NO	NO	35.5	INVALID Test results	NO	0%
		G	YES	YES	32.5	0.4	32.1	33.2	NTRK1-Invalid	YES	100%
44	MET Ex14 Skipping	A	YES	N/A	26.7	N/A	27.7	27.1		YES	100%
		B	YES	N/A	25.9	N/A	27.3	27.4		YES	100%
		F	YES	N/A	27.1	N/A	28.6	30.6		YES	100%
		G	YES	N/A	27.3	N/A	29.3	31.6		YES	100%
45	ROS1 fusion ROS1::SDC4	A	YES	YES	27.6	−1.3	28.8	27.7	ALK exp. Imbl-Invalid	YES	100%
		B	YES	YES	27.9	−1.1	29	29.6		YES	100%
		F	YES	YES	30.2	−1.1	31	32.4		YES	100%
		G	YES	NO	31	−1.1	31.5	32.4	ALK exp. Imbl-Indeterminate	Partial	50%
46	ALK fusion ALK::EML4	F	YES	YES	30.3	1.2	30	32	NTRK2 and NTRK3-Invalid	YES	100%
	RET fusion RET::CCDC6		YES	NO	28.6	−0.6				Partial	50%
	ROS1 fusion SLC34A2::ROS1		YES	YES	31.3	−4.7				YES	100%
47	ALK fusion ALK::EML4	F	YES	NO	31.5	−5.3	32.4	32	ROS1 exp. Imbl/RET exp. Imbl/NTRK1 exp. Imbl not detected (invalid result)	Partial	50%
	RET fusion CCDC6::RET, KIF5B::RET, NCOA4::RET		YES	N/A	31.9	invalid				YES	100%
	NTRK1 fusion LMNA::NTRK1, TFG::NTRK1, TPM3::NTRK1		N/A	N/A	N/A	invalid				N/A	N/A
	NTRK3 fusion ETV6::NTRK3		N/A	YES	N/A	−0.2				YES	100%
	ROS1 fusion SLC34A2::ROS1 CD74::ROS1		YES	N/A	31.2	invalid				YES	100%
	MET Ex14 Skipping		YES	N/A	31.8	N/A				YES	100%
48	ROS1 fusion SLC34A2::ROS1	F	YES	YES	30.8	−3.2	28.6	31.1	NTRK2 and NTRK3-Invalid	YES	100%
49	ALK fusion ALK::EML4	A	YES	YES	25.8	−3.5	25.4	25.9		YES	100%
50	Wild-type Gene fusion not detected	A	YES	YES	N/A	N/A	29.8	30.1	ROS1 exp. Imbl-Invalid	YES	100%

A—FFPE-2 µM slide-scratched; B—HE slides-scratched; F—RNA Input from FFPE scratched slide; G—RNA Input from HE scratched slide; N/A—not applicable; Cq value—quantification cycle; Ex—Exon; exp. Imbl—Expression imbalance.

**Table 6:** Comparative analysis of various sample-type performances in different IVD-molecular assays.

Case No. #	Alternative Method Result	Idylla Cartridge Used	Input Material (s) in Idylla Cartridge	Amount of Input Material	Conclusion of Idylla Test-Mutation Detected YES/NO	Conclusion of Idylla Test − CQ Value-Control/TARGET CQ Value	Overall Concordance	Idylla Performance Analysis
25	BRAF-WT	BRAF	FFPE-HE	2× slides	NO	Invalid	NO	0%
		BRAF	FFPE-2 µM	4× slides	YES	N/A	YES	100%
26	NRAS-WT; BRAF-WT	NRAS-BRAF	FFPE-2 µM	2× slides	YES	34.6	YES	100%
			gDNA-HE	10 µL	YES	38.5	YES	100%
27	EGFR-Ex18:p.G721S	EGFR	FFPE-HE	2× slides	N/A	29.3	N/A	N/A
28	EGFR-Ex19: E746_S752delins	EGFR	FFPE-HE	3× slides	YES	23.7	YES	100%
29	EGFR-Ex21:p.L858R	EGFR	gDNA-FFPE	15 µL	YES	23	YES	100%
30	KRAS-Ex2:p.G12D	KRAS	FFPE-HE + FFPE-2 µM-broth combined	1× HE slide + 2× slides FFPE	YES	20.52	YES	100%
	KRAS-Ex2:p.G12C				Present when raw data was examined. High CQ value −29.3		NO	0%
31	BRAF-Ex15:p.V600E	BRAF	gDNA -HE	10 µL	NO	Invalid	NO	0%
		BRAF	gDNA -HE	25 µL	NO	Invalid	NO	0%
32	EGFR-Ex19:PV742I	EGFR	Cytological fluid	20 µL	N/A	26.7	N/A	N/A
	KRAS-Ex2:G12D	KRAS	Cytological fluid	20 µL and 500 µL	Present when raw data was examined... High CQ value −34.78		NO	0%
	KRAS-Ex2:G12D	KRAS	gDNA from Cytological fluid	40 µL	YES	28.67	YES	100%
	KRAS-Ex2:G12D	KRAS	FFPE-gDNA. Cytoblock	30 µL	YES	25.3	YES	100%
33	EGFR-WT	EGFR	Cytofluid pleural effusion	20 µL	YES	15.9	YES	100%
34	KRAS-Ex2:G12D	KRAS	Cytofluid pleural effusion	500 µL	YES	28.99	YES	100%
	KRAS-Ex2:G12D	KRAS	gDNA from Cytological fluid	40 µL	YES	30.81	YES	100%
35	BRAF-WT	BRAF	FFPE-2 µM	2× slides	YES	N/A	YES	100%
36	MSS	MSI	FFPE-2 µM	2× slides	YES	7/7 System Stabil	YES	100%
37	NRAS-WT; BRAF-WT	NRAS-BRAF	FFPE-2 µM	2× slides	YES	32.7	YES	100%
38	MSS	MSI	HE + PAS-unstained and both combined	2× slides	YES	7/7 System Stabil	YES	100%
39	KRAS-WT	KRAS	FFPE-2 µM	2× slides	YES	N/A	YES	100%
	EGFR -WT	EGFR	FFPE-2 µM	2× slides	YES	18.9	YES	100%
	NRAS-WT; BRAF-WT	NRAS-BRAF	FFPE-2 µM	2× slides	YES	29.3	YES	100%
40	EGFR-Ex20: p.T790M	EGFR	gDNA	10 µL	YES	21.4	YES	100%
	EGFR-Ex19:p.E746_A750del				YES		YES	100%
	EGFR-Ex21:p.L858R				YES		YES	100%
	KRAS- Ex 2:p.G13D	KRAS		10 µL	YES	24.94	YES	100%
	NRAS-WT BRAF-WT	NRAS-BRAF		10 µL	YES	33	YES	100%
	BRAF-WT	BRAF		10 µL	YES	N/A	YES	100%
	MSI-High	MSI		10 µL	YES	4/7 System unstable	YES	100%

FFPE—formalin-fixed paraffin-embedded; gDNA—Genomic DNA; N/A—not applicable; NE—not evaluated; Ex—Exon; WT—wild-type; conc—concentration; MSI—microsatellite instability; MSS—microsatellite stable; HE—hematoxylin-eosin-stained slides.

## Data Availability

Not applicable.

## Supplementary Materials

The following supporting information can be downloaded at: https://www.mdpi.com/article/10.3390/ijms232012515/s1.
