# Supplementary material for: Assessing and Evaluating the Scope and Constraints of Idylla Molecular Assays by Using Different Source Materials in Routine Diagnostic Settings
Source: Int J Mol Sci. 2022 Oct 19;23(20):12515. doi: 10.3390/ijms232012515 (PMC9604085; doi:10.3390/ijms232012515)
Supplement: Supplementary file 1 [file ijms-23-12515-s001.zip › ijms-1964041-supplementary.pdf]

Supplementary Materials

Table S1. Testing the repeatability of all Idylla assays for different sample types (A-G).

	Conclusion of Idylla Test - Cq Value - EGFR Control/ NRAS Control/ KRAS Target Cq Value/ BRAF Target Cq Value					
**Sample Type**	**RUN 1**	**RUN 2**	**RUN 3**	**Mean**	**STD DEV**	**Coefficient of variation (CV%)**
SAMPLE TYPE A						
EGFR	19.9	19.7	19.6	19.73	0.15	0.76
BRAF	33.56	33.47	33.68	33.57	0.1	0.29
KRAS	23.98	23.65	23.79	23.8	0.16	0.67
NRAS-BRAF	41.82	41.68	41.58	41.69	0.12	0.28
MSI	7	7	7	7	0	0
Genefusion	27.7	27.4	27.5	27.53	0.15	0.54
SAMPLE TYPE B						
EGFR	19.5	20.2	21	20.23	0.75	3.7
BRAF	37.57	38.45	37.89	37.97	0.44	1.15
KRAS	29.45	29.56	30.5	29.83	0.57	1.91
NRAS-BRAF	44.82	42.68	41.58	43.02	1.64	3.81
MSI	6	6	6	6	0	0
Genefusion	26.1	25.6	26.7	26.13	0.55	2.1
SAMPLE TYPE C / F						
EGFR	20.2	20.5	20.8	20.5	0.3	1.46
BRAF	34.57	35.01	34.89	34.82	0.22	0.63
KRAS	25.57	25.12	25.32	25.33	0.22	0.86
NRAS-BRAF	42.36	41.97	42.62	42.31	0.32	0.75
MSI	7	7	7	7	0	0
Genefusion	29.4	29.8	29.6	29.6	0.2	0.67
SAMPLE TYPE D / G						
EGFR	22.6	23.1	23.5	23.06	0.45	1.95
BRAF	37.77	38.21	37.52	37.83	0.34	0.89
KRAS	31.68	31.82	31.24	31.58	0.3	0.94
NRAS-BRAF	43.3	44.5	43.9	43.9	0.6	1.36
MSI	7	7	7	7	0	0
Genefusion	26.8	27.1	26.5	26.8	0.3	1.11

A= FFPE -2μM slide- scratched; B= HE slide- scratched; C= DNA Input from FFPE scratched slide; D= DNA Input from HE scratched slide; F= RNA Input from FFPE scratched slide; G= RNA Input from HE scratched slide; N/A=not applicable; Cq value=quantification cycle						

Table S2. Testing the performance of the archival tissue samples in 2μM FFPE tissue sections (Sample type A). List of the additional samples tested along with the others mentioned in the results table 1 and 2.

Case #	Tissue preparation year	Tumor cells (%)	Cancer type	Previous Result	Idylla cartridge used	Idylla result- concordance
S1	2013	95%	NSCLC	EGFR - Ex21:p.L858R	EGFR	YES
S2	2014	70%	NSCLC	EGFR - Ex 19: p. E746_S752delinsA	EGFR	YES
S3	2012	85%	NSCLC	EGFR-WT	EGFR	YES
S4	2015	75%	Melanoma	BRAF-Ex15:p.V600E, NRAS-WT	NRAS/ BRAF	YES
S5	2014	70%	CRC	NRAS -Ex 3:p.Q61R<br>BRAF-WT	NRAS/ BRAF	YES
S6	2005 (MTB)	60%	CRC	KRAS- Ex2:p.G12C<br>KRAS- Ex2:p.G12D	KRAS	YES
S7	2009	50%	CRC	KRAS- Ex 2:p.G13D	KRAS	YES
S8	2013	70%	NSCLC	KRAS- Ex 2:p.G12C	KRAS	YES
S9	2014	80%	Thyroid cancer	BRAF-Ex15:p.V600E	BRAF	YES
S10	2016	85%	Melanoma	BRAF-Ex15:p.V600E	BRAF	YES
S11	2011	70%	CRC	MSI High (5/10 System instable)	MSI	YES
S12	2010	80%	CRC	MSS	MSI	YES
S13	2011	90%	CRC	MSS	MSI	YES
S14	2010	80%	NSCLC	Fusions - Wildtype	Gene fusion	YES
S15	2012	90%	NSCLC	ALK fusion ALK::EML4	Gene fusion	YES

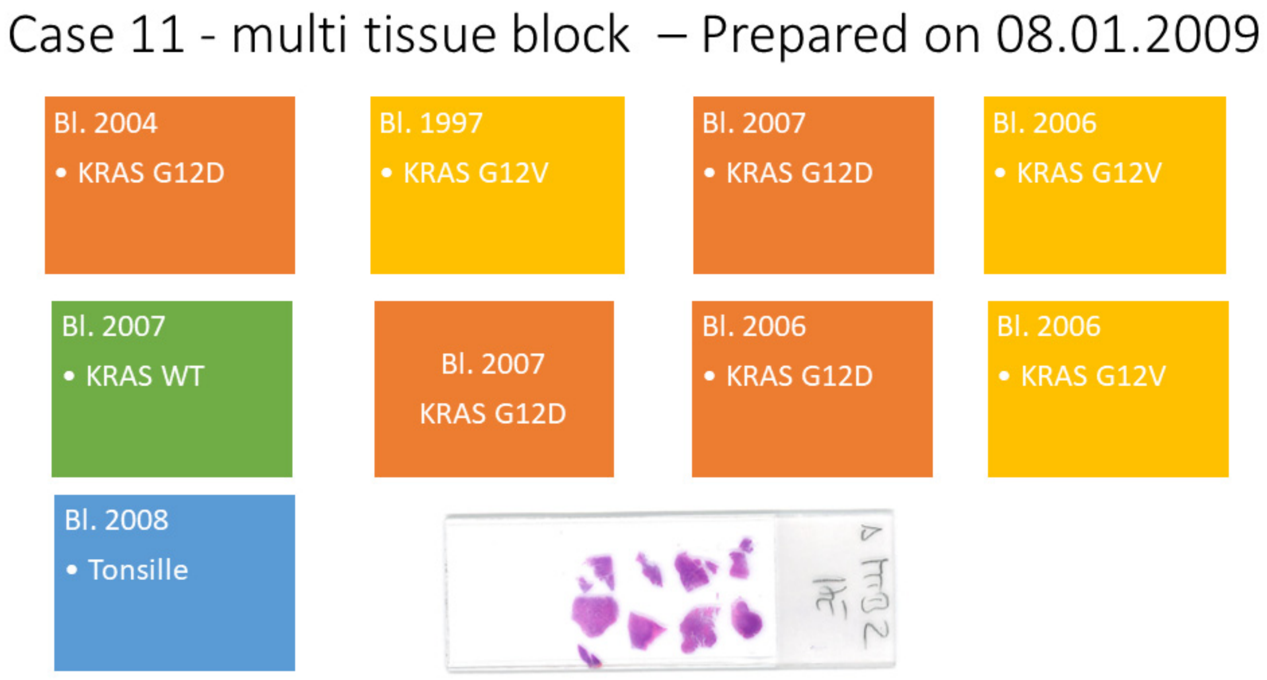

Figure S1. Case 11- schematic representation of the multi tissue section of case 11 with their respective KRAS mutation depictions with the year of the prepared original FFPE block. The HE stained slide of the MTB is also presented for reference.
